# Effect of manual versus mechanically assisted manipulations of the thoracic spine in neck pain patients: study protocol of a randomized controlled trial

**DOI:** 10.1186/s13063-015-0763-5

**Published:** 2015-05-27

**Authors:** Anke Langenfeld, B. Kim Humphreys, Rob A. de Bie, Jaap Swanenburg

**Affiliations:** Department of Chiropractic Medicine, University of Zurich and Balgrist University Hospital, Forchstrasse 340, CH-8008 Zurich, Switzerland; Department of Physiotherapy, Balgrist University Hospital, Zurich, Switzerland; Department of Epidemiology and CAPHRI School for Public Health and Primary Care, Maastricht University, Universiteitssingel 60, NL-6229 Maastricht, ER The Netherlands; Physiotherapy Occupational Therapy Research, Directorate of Research and Education, University Hospital Zurich, Zurich, Switzerland

**Keywords:** Neck pain, Spinal manipulation, Impulse iQ®, Thoracic spine

## Abstract

**Background:**

Neck pain is a common musculoskeletal condition with a point prevalence of around 15 % in males and 23 % in females that often presents in physiotherapy practice.

Physical therapy and/or manipulation therapy is generally the first management option for patients with mechanical neck pain. Physical therapists treat mechanical neck pain with a number of interventions including joint mobilization and/or manipulation, therapeutic exercises or education. However, manipulation of the cervical spine carries some risks. Treating the thoracic spine for neck pain is an alternative approach. Emerging evidence suggests that it may be effective for treating neck pain without the risks associated with cervical spine manipulation. A new electromechanical device has recently been developed and tested for delivering multiple high velocity, low amplitude thrust manipulations to the spine. This device incorporates both auditory and visual systems that provide real time feedback on the applied treatment. The objective of this study is to compare the short- and long-term effects of manual versus mechanically assisted manipulations of the thoracic spine for neck pain patients.

**Methods/Design:**

A 6-month, randomized controlled trial consisting of 54 patients with acute or chronic neck pain patients will be conducted. Patients with no signs of major pathology and with little or no interference with daily activities will be recruited. Three treatment sessions with 4-day intervals will be carried out. The patients will be randomly assigned to receive either manually performed manipulations or electromechanical manipulations at the thoracic spine. The primary outcome is pain intensity as measured by the Visual Analogue Pain Rating Scale. The secondary outcome measures are neck physical disability using the Neck Disability Index, quality of life measured by the European Quality of Life 5 Dimensions 5 Levels and patients’ improvement using the Patient’s Global Impression of Change Scale.

**Discussion:**

It is expected that both interventions will improve neck pain. This would be a significant finding, as thoracic spine manipulation for neck pain does not carry the same risk of injury as cervical spine manipulation. In addition, the results may provide useful information about therapeutic options for health care providers and patients for the problem of neck pain.

**Trial registration:**

Current Controlled Trials ISRCTN88585962, registered January 2013

**Electronic supplementary material:**

The online version of this article (doi:10.1186/s13063-015-0763-5) contains supplementary material, which is available to authorized users.

## Background

The burden of musculoskeletal disorders is increasing rapidly, using a high percentage of health care resources [1]. Neck pain is a musculoskeletal disorder and is a common cause for patients seeking treatment from health care providers [2]. Most people will see a medical practitioner or another health care provider at least once in their lifetime due to neck pain [1]. Alarmingly, 30 to 50 % of the general population suffer from neck pain annually, with a point prevalence of around 15 % in males and 23 % in females; and these numbers are increasing [1, 3]. Even more concerning is the fact that someone who has experienced one episode of neck pain is likely to have another episode within the next 1 to 5 years [4], and can be expected to suffer for several years, with a low chance of full resolution.

Neck pain is generally described as pain perceived in the posterior region of the cervical spine. It is distributed between the superior nuchalline down to the first thoracic spinous process and may radiate into the head, shoulders, arms and chest [5]. The Task Force on Neck Pain and its Associated Disorders proposed the following classification for neck pain patients:

- Grade I: no signs of major pathology and little or no interference with daily activities. This is frequently the case
- Grade II: no signs of major pathology, but with an interference with daily activities. This occurs less frequently
- Grade III: neck pain with neurological signs or symptoms (radiculopathy)
- Grade IV: neck pain with signs of major pathology (for example, serious instability or spinal infection) [6]

Current opinion regarding the etiology and onset of neck pain and how it is best managed varies widely, and often seems to be associated with the background and beliefs of the clinician [6, 7]. Treatments that have been recommended for neck pain within physical therapy settings so far are exercise therapy, manipulation therapy and a combination of exercise and cervical spinal manipulation as well as education: they seem to have the highest level of evidence [8]. There are also other treatment options: for example, acupuncture, cognitive/behavioural therapy, and electro-physical modalities, but so far there is conflicting evidence about their benefit [8].

Although cervical spinal manipulation is still recommended for the treatment of neck pain, there is a small risk of possible serious side-effects such as cervical artery dysfunction (CAD), lesions of the brain stem, Wallenberg syndrome or ischemic stroke [9–11]. Although these presentations are rare they should be considered as part of the patient’s assessment [11]. The literature is equivocal on a link between the use of cervical spinal manipulation and serious risks [9, 10, 12–14]. Studies show no clear relationship between cervical spinal manipulation and an arterial dissection although the manipulation process is capable of triggering such an event, because cervical spinal manipulations are comparable to minor mechanical events, which might cause arterial dissection [15, 16]. Furthermore, literature suggests that the dissection of the vertebral or carotid artery may have already been in progress before the manipulation is given due to the fact that the dissection causes pain similar to mechanical neck pain and, therefore, patients visit a chiropractor or a primary care physician because of these complaints [17, 18]. However, there is still a debate in the current research whether manipulation of the cervical spine in patients with neck pain is harmless [10].

Pre-manipulative testing and screening procedures were introduced to minimize the risk of side-effects in cervical spinal manipulations [10]. Unfortunately these procedures have shown a lack of sensitivity, specificity, and feasibility [8, 10, 15, 17, 18]. To address the issues of the risk of a serious adverse event and a lack of useful testing/screening procedures, a different treatment strategy has been utilized [19]: spinal manipulation directed at the thoracic spine instead of to the cervical spine [20]. The justification for using thoracic spinal manipulations for the treatment of neck pain is based on the biomechanical link between cervical and thoracic spine [21–23]. Normal functioning of the cervical spine depends on normal biomechanics of the upper thoracic spine. If there is any functional disturbance in the upper thoracic spine, the capacity of the muscles decreases and the range of motion of the cervical spine is impaired [23].

Within the last few years several intervention studies, systematic reviews, and meta-analyses have been conducted investigating thoracic spinal manipulations [20, 24–30]. The majority of these intervention studies were focused on patients with acute or a primary complaint of neck pain and they reported short-term outcomes [20, 24, 26]. Thus far, only 1 study has investigated patients suffering from chronic neck pain over a long-term outcome of 6 months [27]. Nevertheless, the Orthopaedic Section of the American Physical Therapy Association (APTA) included the use of thoracic spinal manipulations as a possible treatment option in their guidelines for the treatment of patients with neck pain and neck-related arm pain, although this is based on weak evidence [25]. Young and colleagues (2014) state that there is a significant amount of evidence to recommend thoracic manipulation for the treatment of mechanical neck pain, especially for short-term improvement of range of motion and disability [31].

A manipulation may be delivered manually (high velocity, low amplitude) or mechanically using spring-loaded or electromechanical devices (mechanical force, manually assisted) [32–35]. Forces applied during a manual spinal manipulation may vary somewhat from manipulation to manipulation given by one practitioner as well as between practitioners [36]. Therefore, a mechanical instrument may have less force variation and, therefore, more consistency [32]. Mechanical devices may be useful in reducing the variations in the practitioner’s performance of spinal manipulation [34]. Two randomized controlled trials have been conducted so far, comparing the outcome of manual and mechanically delivered manipulations in patients with neck pain and patients with sacroiliac joint pain [34, 35]. One study used manual manipulations compared to mechanical force, using a spring-loaded device. The manipulations were applied to the cervical spine [34]. The other study also compared manual manipulation to a mechanical-force, manually-assisted instrument (Activator Adjusting Instrument) (Activator Methods International, Ltd, Phoenix, Arizona, USA). In this study the manipulations were applied to the sacroiliac joint [35]. Both studies concluded that both methods of applying manipulations are beneficial for the patient in terms of pain reduction, range of motion and disability of the treated area [34, 35]. Although there are only two studies using different body regions and mechanical and manual manipulations in comparison, both ways of applying a manipulation seem to be beneficial.

**Fig. 1:**
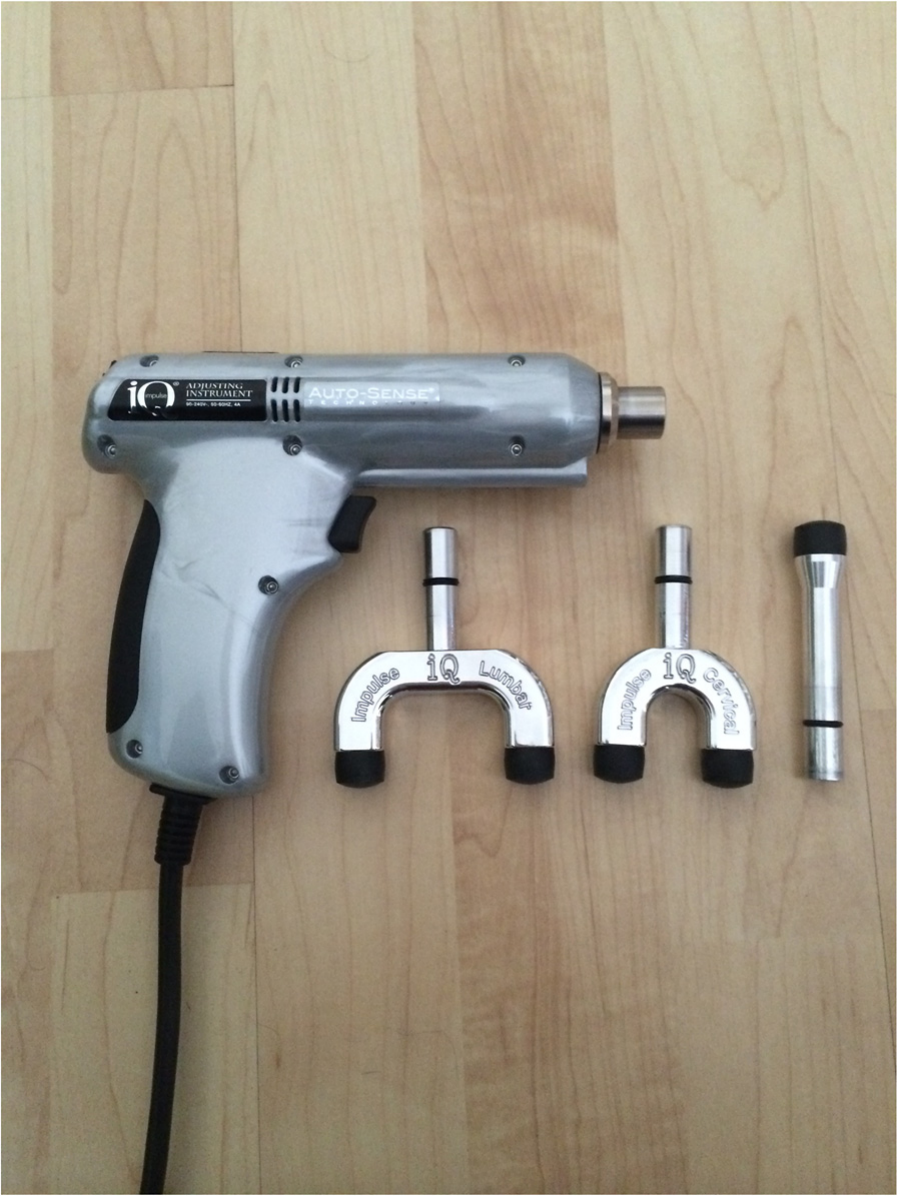
Impulse IQ®. Picture of the device that was used to conduct the mechanical manipulations

A new electromechanical device for manipulations has recently been developed, which purports to give objective feedback to the practitioner, using an audible sign (beep) (Fig. 1). The device contains a motion sensor and a microcomputer. While the thrusts are delivered, the amount of spinal motion and the frequency of motion are identified in real time. As the vertebra rebounds, data is sent to the microcomputer. Auto-Sense Technology® (Neuromechanical Innovations, Chandler, AZ, USA) consequently adapts the amount of the subsequent thrusts. If the acceleration response has been maximized, the treatment stops automatically [37]. However, the exact effect remains unclear if this electromechanical device does what it purports to do, and if it has the same effect as manually performed manipulations.

Therefore, the purpose of this study is to compare the effects of manually performed manipulations and electromechanical manipulations to the thoracic spine in neck pain patients. Short- and long-term clinical outcomes, such as neck pain and function and overall improvement, will be assessed.

### Study aim

The aim of this study is to compare the short and long-term treatment outcomes for neck pain patients using an instrument-assisted mechanical manipulation with feedback versus manual manipulations of the thoracic spine.

## Methods/Design

This study is a randomized trial comparing the treatment effects of manual versus instrument- assisted mechanical manipulations of the thoracic spine for neck pain patients. The study has received ethical approval from the Ethics Commission of the Canton of Zurich (2012-0248).

### Enrollment and eligibility criteria

Participants will be recruited by registered chiropractors and physical therapists in private practices in the canton of Zurich. A test group of 54 participants will be recruited based on the following inclusion criteria: 1) presence of acute or chronic neck pain, Grade I or II [6]; 2) aged 18 or older; 3) able to speak and read German or English; 4) no previous manual therapy applied to the thoracic spine; and 5) interested in participating in the study. Potential participants will be excluded from the study if they suffer from the following conditions: 1) severe disorders of the cervical spine such as disc prolapse, spinal stenosis, postoperative conditions in neck and shoulder areas; 2) history of severe trauma; 3) spasmodic torticollis; 4) frequent migraine headaches; 5) peripheral nerve entrapment; 6) fibromyalgia; 7) shoulder diseases (causing reduced mobility of the joint: for example, fractures, adhesive capsulitis); 8) inflammatory rheumatic diseases; 9) osteoporosis; 10) cancer and 11) be currently undergoing legal procedures resulting from their neck pain.

If and when the participant qualifies based on the above criteria and has signed the informed consent form, the recruiting chiropractor or physical therapist will inform the research assistant of their suitability for the study. The research assistant will verify the inclusion and exclusion criteria and invite the participant for the baseline assessments.

### Design

**Fig. 2:**
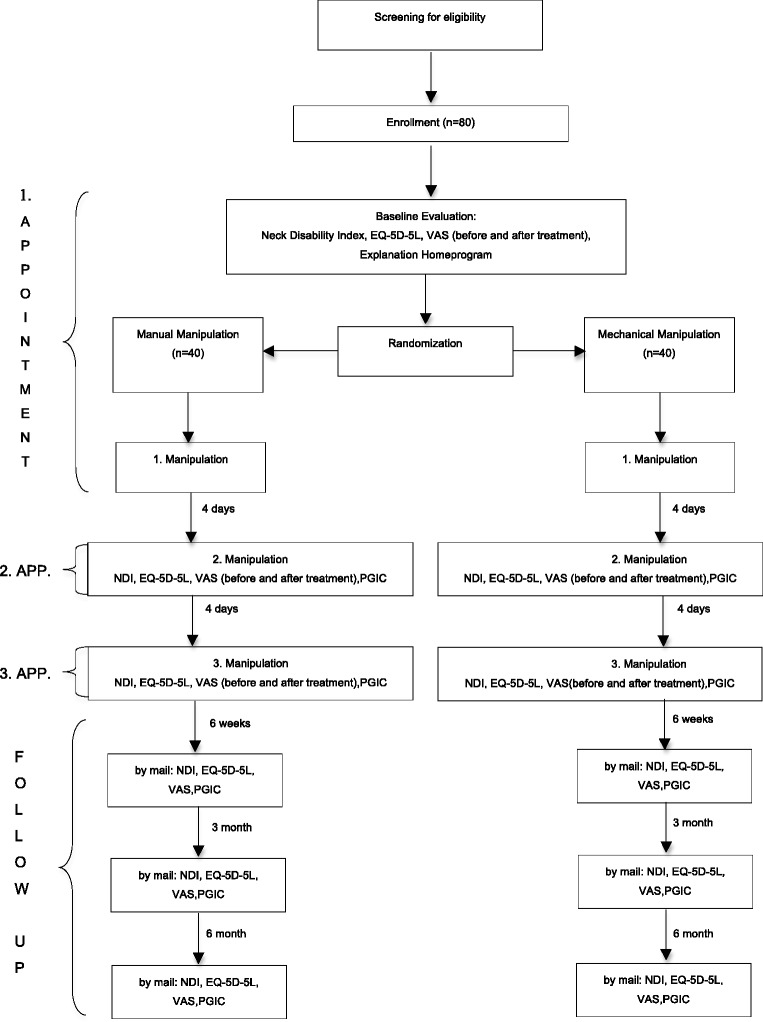
Flowchart of the trial. Flowchart of the trial showing how the participants were allocated to the different groups and the follow-up

There will be 3 treatment sessions and an additional training program for 6 weeks. Follow-up assessments will take place after 6 weeks, 3 months and 6 months. The 3 treatment session will always be 4 days apart. At baseline the participant will fill out the Visual Analogue Pain Rating Scale (VAS), the Neck Disability Index (NDI) and the European Quality of Life 5 Dimensions 5 Levels (EQ-5D-5 L) [38–45]. At two subsequent treatment sessions, the patient will be asked to fill out the NDI, VAS, EQ-5D-5 L, and the Patients Global Impression of Change Scale (PGIC) before the treatment [46]. The VAS will be repeated after each treatment. For the follow-up assessments the participants will receive the same questionnaires (Fig. 2).

### History taking and physical examination

Baseline measurements as stated above, will be followed by a history taking and physical examination (P/E). History taking includes questions referring to the onset, episode, nature and location, as well as factors that increase or decrease the pain, medication use, occupation, sick leave and other treatments that have already been tried. The P/E will start with a neurological screening (motor, reflex and sensory testing) followed by Spurling’s test, cervical distraction test and neurodynamic upper limb test [47–50]. These evaluations are added to confirm that the patient is eligible to participate in the study. If one of the previous tests is positive the patient will be excluded from the study at this point. Next the segmental mobility of the entire thoracic spine will be tested. In this study a dysfunctional segment is defined as a painful segment, that is produced using prone springing palpation for pain provocation [51, 52]. We define the painful segment as the treatment segment [53]. The testing and marking of the thoracic spine will be carried out by an orthopedic manual therapy (OMT)-trained physical therapist, who will also be applying the manipulations. The most painful segment is marked with a waterproof marker and then photographed; followed by the randomized treatment of the painful thoracic segment. Other painful and/or hypomobile segments will be marked and reported but they will not be treated.

### Randomization

A clinician, who is not involved in the study, will independently conduct the randomization procedure. A block randomization (20 blocks of 4) using a computer-based randomization program will be conducted. The original randomization list will be stored in an opaque and sealed envelope not accessible to the therapist conducting the manipulations. Eighty sequentially numbered, sealed, opaque envelopes, containing the randomized group allocation will be prepared. At the time of treatment, these envelopes will be given to the therapist applying the manipulation, immediately prior to the execution of the manipulation. The patient will be unaware of the technique that will be applied, only that the technique may either be delivered manually or with an electromechanical instrument (Impulse iQ®, Neuromechanical Innovations, Chandler, AZ, USA). To minimize any possible effects of expectations of treatment, each participant will be asked prior to the first treatment about their expectations regarding new and conventional treatments.

### Blinding

To minimize performance bias, the therapist who is conducting the treatment will be kept unaware of the treatment method until the manipulation is applied. Additionally, the questionnaires that are filled out by the patient are put inside sealed, opaque envelopes. The therapist is unaware of the patient-assessed outcome. When data is entered into the database the researcher will be unaware of the associated participant due to de-personalization/anonymity of the questionnaires.

Additionally, the expectations of the patient regarding the new and conventional treatments are retrieved beforehand and taken into consideration during the evaluation of the data.

### Treatment

After the initial P/E the first manipulation will be applied to the thoracic spine either manually or by using the Impulse iQ® (Neuromechanical Innovations, Chandler, AZ, USA) and the training program, consisting of four different exercises, will be instructed.

### Manual

**Fig. 3:**
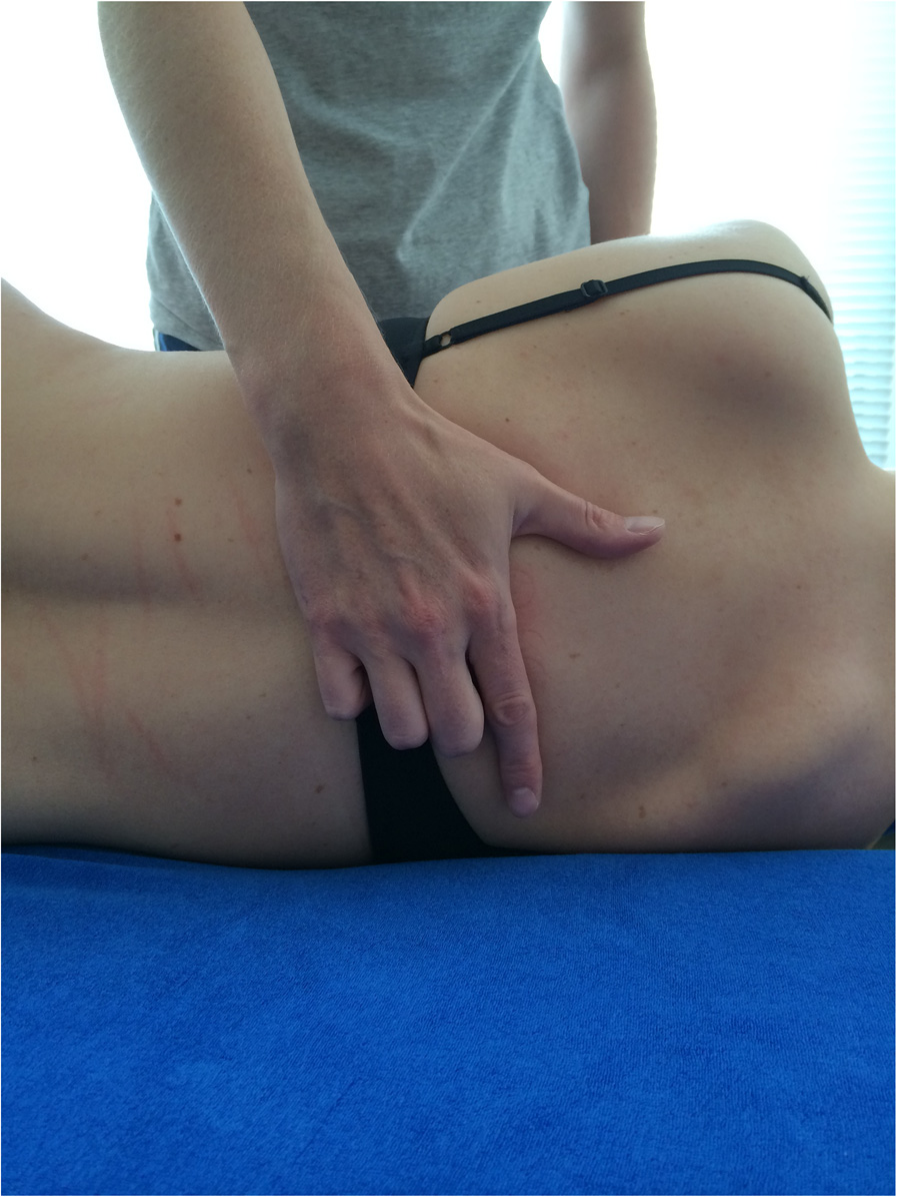
Hand position. This picture shows how the hand of the therapist has to be positioned during the manual manipulation

**Fig. 4:**
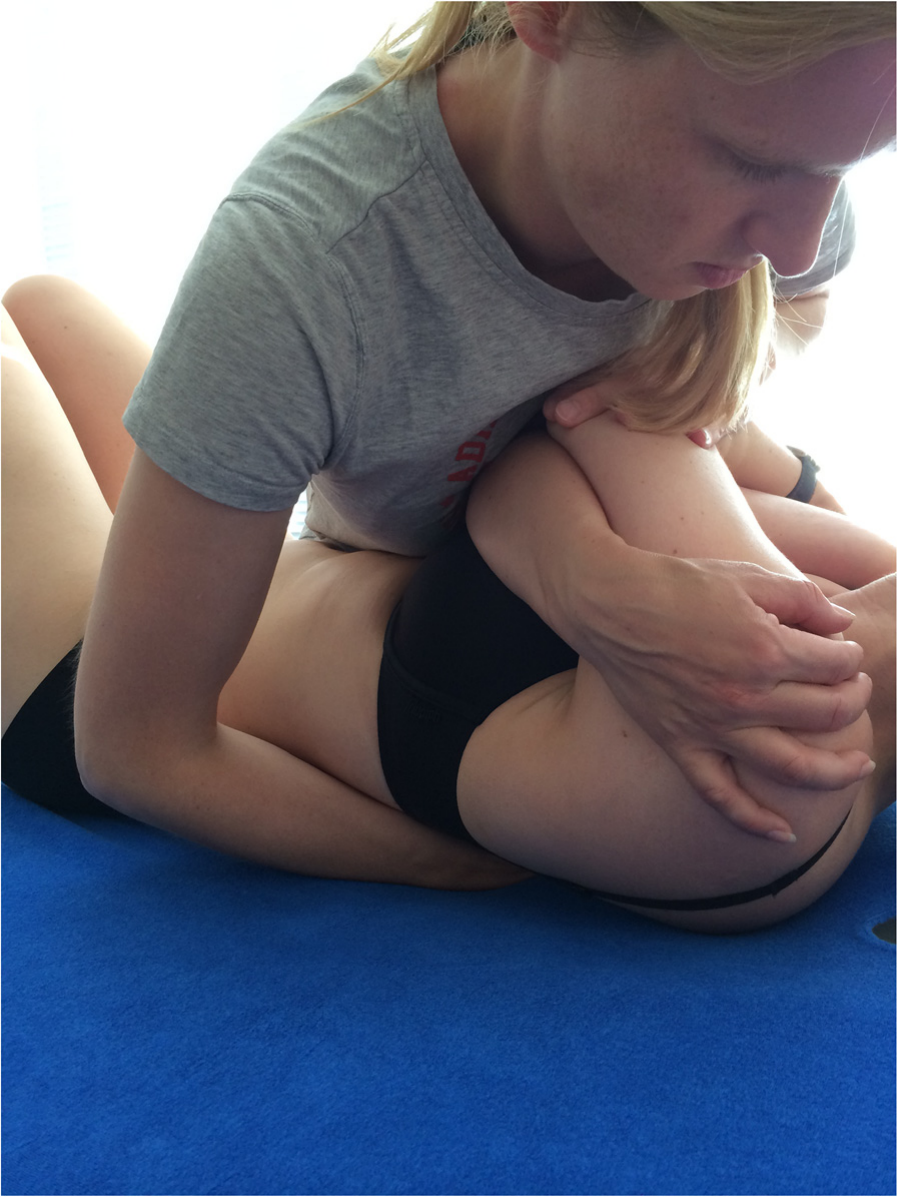
Manual manipulations. Positioning of the patient and therapist’s body during the manual manipulation

For the manually performed manipulations the patient is lying supine. The therapist’s hand is under the thoracic spine using a pistol grip (the fingers are positioned with the index finger straightened and fingers 3 to 5 flexed). The patient’s forearms are crossed in front of their chest. The force is applied against the therapist’s flexed fingers, the thenar eminence and slightly cranial to the transverse process of the caudal vertebra of the treatment segment [53] (Figs. 3 and 4).

### Mechanical

**Fig. 5:**
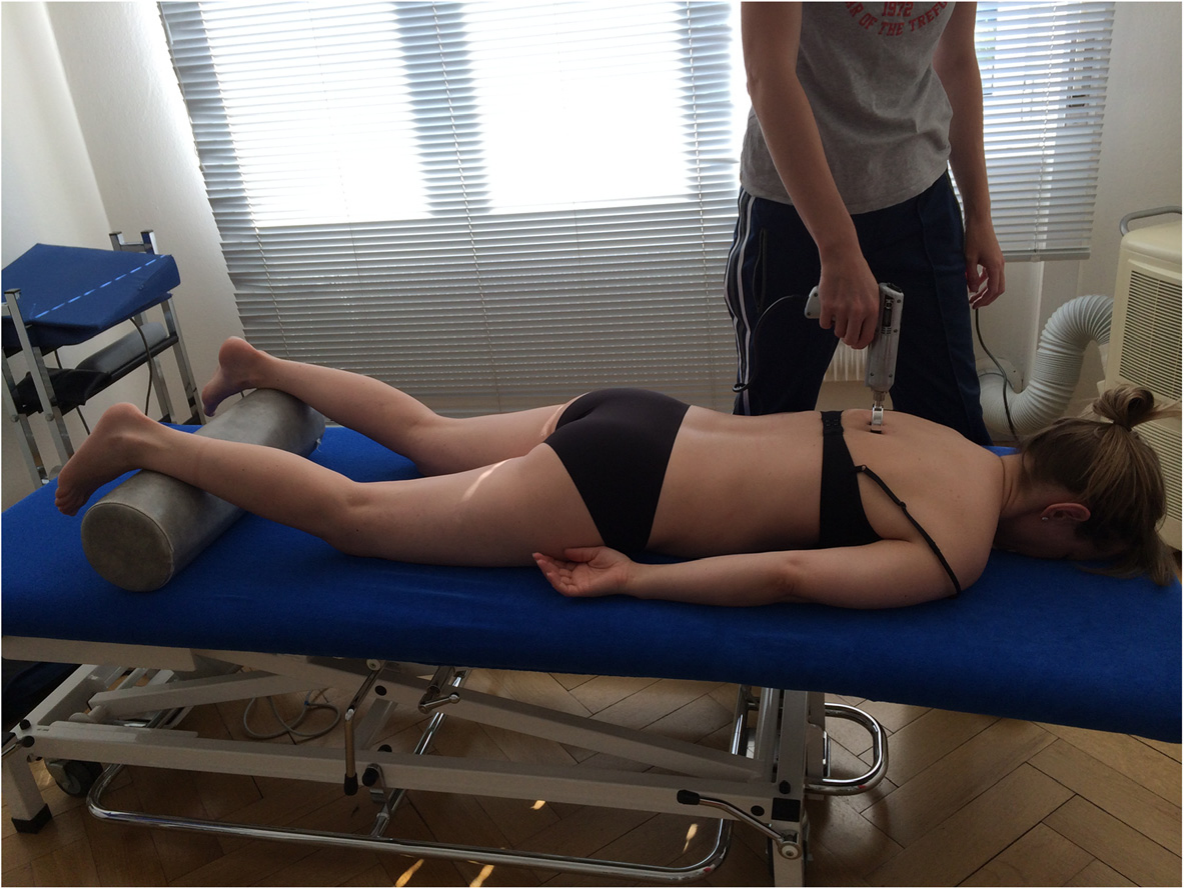
Patient position during mechanical manipulation. The patient lies prone, arms next to the body in a relaxed position

**Fig. 6:**
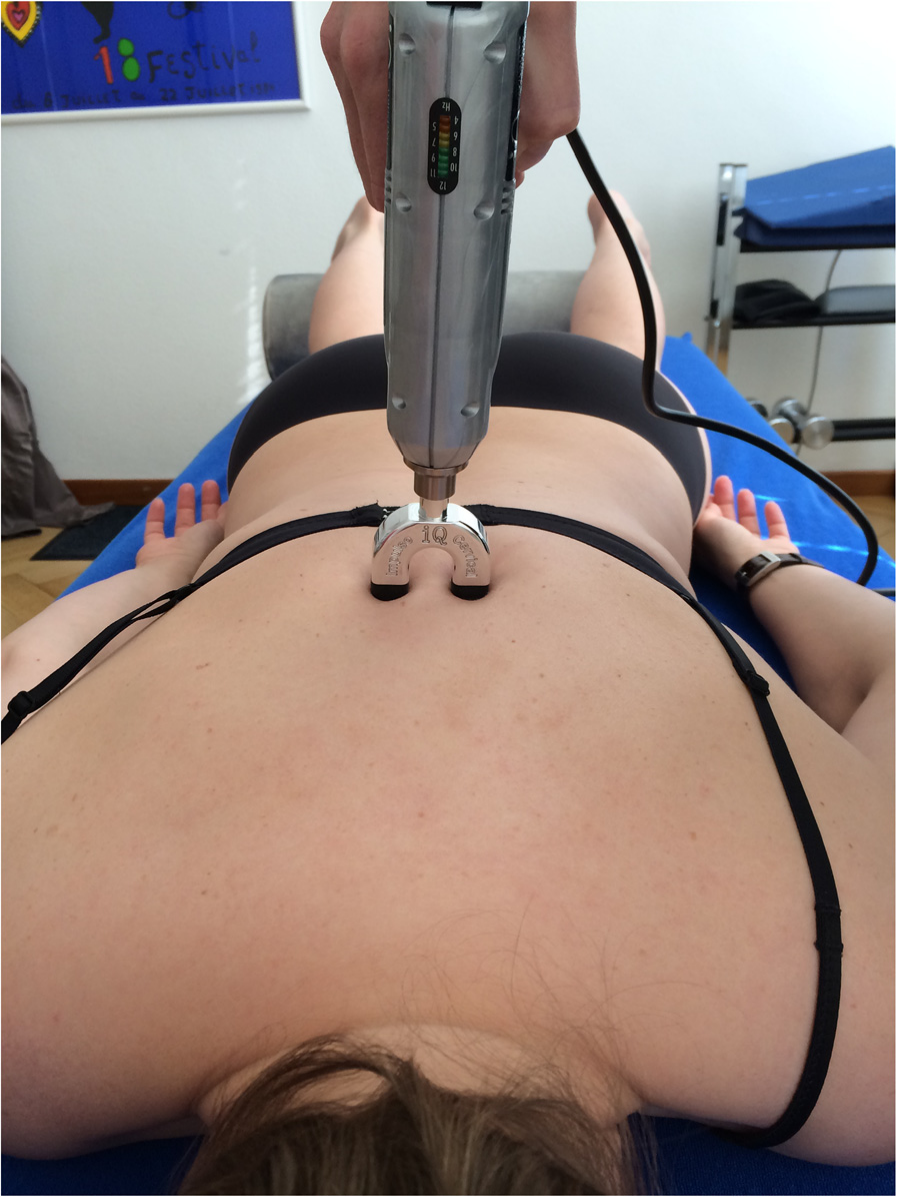
Positioning of the double stylus. The double stylus is positioned directly on the treatment segment

For the Impulse iQ® (Neuromechanical Innovations, Chandler, AZ, USA) the patient lies prone on a treatment table, with arms next to the body, in a relaxed position. Before the treatment begins, the patient is instructed that they will hear a rattling sound that indicates the thrusts conducted by the device and a beep at the end of the treatment. The Impulse iQ® (Neuromechanical Innovations, Chandler, AZ, USA) is then put onto the vertebra that has been identified as a painful segment. A double stylus and middle force setting (peak force = 200 N), as recommended by the manufacturer for the treatment of the thoracic spine, will be used [54]. The device records and analyzes the spinal acceleration response each time a thrust is delivered using the built-in firmware. The machine then produces a series of repetitive thrusts monitoring the acceleration response and, if the response is improving, treatment continuous up to 3 s. If the acceleration response is negative (flat line or decrease) the thrust delivered by the instrument ceases (Figs. 5 and 6).

### Training program

After the first treatment session the patients will all be introduced to the home exercise program and asked to perform this program once a day for 6 weeks, while compliance with the home exercise program is assessed using a training journal:

1. Retraining the cranio-cervical muscles
  - Training the holding capacity of the deep neck flexors [55]
  - Head Lift [56]
2. Retraining scapular muscles [55]
3. Eye/Head coordination [55]

A detailed exercise-program may be found in Additional file 1.

### Outcomes

#### Primary outcome measure

The primary outcome measure is the VAS before and after every treatment, after 6 weeks, 3 months and 6 months. The VAS has shown to be a valid and reliable outcome measure [38–41].

The VAS tries to assess a person’s pain intensity level. The patient is asked to place a mark on a 100-mm long line with the question ‘how much pain do you have at this moment’. The beginning of the line illustrates ‘no pain’ the end of the line ‘extreme pain’. To extract the data the researcher measures the distance in millimeters between ‘no pain’ and the mark set by the participant.

#### Secondary outcome measures

Secondary outcome measures are the NDI, EQ-5D-5 L and the PGIC.

The German version of NDI is a questionnaire to assess self-rated disability in neck pain patients. It is a short paper-pencil instrument, which is based on a similar instrument used for patients with lower back pain, the Oswestry Low Back Pain Questionnaire. It consists of ten items: for example, working and driving. Each item has a score up to 5 with a total score of 50. The lower the score the less the self-rated disability [42, 43].

The EQ-5D-5 L is a valid and reliable self-report questionnaire that measures patient’s health status for clinical and economic appraisal using a descriptive system and a VAS [44, 45]. The descriptive system assesses mobility, self-care, usual activities, pain, discomfort and anxiety/depression and patients choose the most appropriate option from ‘no problem’ to ‘extreme problem’. The digits for the five dimensions can be combined in a five-digit number, which describes the health status. The VAS is vertical and 20 cm long and the endpoint options range from ‘the best health you can imagine’ to ‘the worst health you can imagine’. The participant is asked to mark the scale with a ‘X’ and write the corresponding number in a box [57].

The PGIC is a valid outcome measure that is based on a seven-point Likert scale [58]. The purpose of the PGIC is to obtain a patient’s report of their improvement over time during treatment. The scale ranges from ‘much better’, ‘better’, ‘somewhat better’, ‘no change’, ‘somewhat worse’, ‘worse’ and ‘much worse’. ‘Much better’ is rated as 7 and ‘much worse’ as 1 on the PGIC [59]. With this scale it is possible to dichotomize the participants into 2 groups; namely those that have ‘improved’ (ratings of 6 and 7) and those that have ‘not improved’ (ratings 1 to 5).

### Statistical methods

#### Number of test subjects

The sample size was calculated with the formula:

$$ \mathrm{n} = \mathrm{f}\left(\upalpha, \upbeta /2\right)*2*{\upalpha}^2/{\mathrm{d}}^2 $$

Therefore: 2 × [(1.96 + 0.842)^2^ × 15^2^]/12^2^ = 24.53 [60]

The significance level alpha was chosen at 5 % and the power of 80 %.

From the literature, an expected standard deviation of 15 mm of a VAS can be determined [19]. For the expected mean difference the minimal clinically significant difference (12 mm) on the VAS was determined [61]. To be able to accomplish all needed participants, 2 patients per group will be added addressing possible withdrawals, missing data, and losses to follow up [62]. Therefore, a total of 54 patients will be recruited (27 per arm).

### Statistical analyzes

Descriptive statistics will be used to illustrate the empirical data and research population. The Shapiro-Wilk test will be used to assess whether all the variables are normally distributed. If the data is normally distributed the changes of VAS will be analyzed using analysis of variance (ANOVA) (repeated measures) to explore possible significant interaction (group x time) effects (α[1]0.05). Secondary outcome variables will be compared using the Wilcoxon test for matched pairs including confidence intervals. Participants will be sub-grouped into acute and chronic to compare the outcomes. To analyze the outcome of the PGIC, patients will be split into 2 groups (dichotomized): improved (6 and 7) and not improved (1 to 5). With the help of logistic regression, the predicted probability of the outcome will be assessed using the PGIC as dependent variables (improved/not improved). Independent variables will be age, sex, expectations, manipulation and duration of complaints. Missing data will be analyzed using an intention-to-treat method. The data will be collected, stored and analyzed in the IBM-SPSS 22 (PASW, Chicago, IL, USA) statistics program.

### Ethics and data security

This trial has been approved by the Ethics Committee of the Canton of Zurich (KEK-ZH-number 2012-0248). All patients will be asked to provide written informed consent prior to participating in this study. This trial is registered at Current Controlled Trials (ISRCTN 88585962). The collected data will be locked in a secure cabinet and saved at Balgrist University Hospital for 20 years.

## Discussion

The purpose of this study is to compare treatment outcomes for neck pain patients using manual versus instrument-assisted mechanical manipulation at the thoracic spine. For this aim, a randomized clinical trial is the appropriate study. There are already existing trials and reviews that have investigated the treatment of the thoracic spine in neck pain patients [20, 24–30]. Although there are trials that have compared mechanical and manual manipulations [34, 35], no study has worked with the Impulse iQ® (Neuromechanical Innovations, Chandler, AZ, USA). There are several factors which will support the outcome of the study. These are based on the latest knowledge about the treatment of neck pain. First, patients will receive a treatment using an interval of 4 days between each manipulation. This is recommended in the clinical prediction guideline rules for the use of thoracic manipulations in neck pain patients. Second, we added a home exercise program. It is recommended to combine manipulations of the spine with exercises, to have the strongest benefit for patients with neck pain [8]. Third, all outcome measures that are used (VAS, NDI, EQ-5D-5 L) are valid and reliable and have been often used in research. Fourth, to minimize selection bias, a person not involved in the study conducted the randomization of the participants using a computer program. The outcome of the data sheet was then written on cards and put inside opaque, sequentially numbered and sealed envelopes.

One of the trial’s weaknesses might be regarding blinding. We addressed this issue in different ways. To minimize performance bias, the therapist who is conducting the treatment will be unaware of the treatment method until the manipulation is applied. Additionally, the filled-in questionnaires are put inside a sealed, opaque envelop by the patient. The therapist is unaware of the outcome. When the data is put into the database the therapist will be unaware of the associated participant due to de-personalization/anonymity of the questionnaires. Additionally, the expectations of the patient regarding the new and conventional treatment are retrieved beforehand and taken into consideration during the evaluation of the data.

The results of this study may provide useful information for clinicians and patients in terms of effective therapeutic options for treating neck pain patients without the risk of cervical spine manipulation. The outcomes of the research will be published in a timely manner after the completion of this study**.**

## Trial status

Recruitment of study participants is currently ongoing.

### Consent section

Written informed consent was obtained from the patient(s) for publication of this manuscript and accompanying images. A copy of the written consent is available for review by the Editor-in-Chief of this journal.

## Additional file

Additional file 1:
**Exercise program: neck.**

## Abbreviations

ANOVA: analysis of variance
APTA: American Physical Therapy Association
CAD: cervical artery dysfunction
EQ-5D-5 L: EuroQol 5 Levels 5 Dimensions
NDI: Neck Disability Index
OMT: orthopedic manual therapy
P/E: physical examination
PGIC: Patients’ Global Impression of Change Scale
VAS: Visual Analogue Pain Rating Scale
