# Supplementary material for: Effect of manual versus mechanically assisted manipulations of the thoracic spine in neck pain patients: study protocol of a randomized controlled trial
Source: Trials. 2015 May 27;16:233. doi: 10.1186/s13063-015-0763-5 (PMC4464708; doi:10.1186/s13063-015-0763-5)
Supplement: Additional file 1: — Exercise program: neck. [file 13063_2015_763_MOESM1_ESM.pdf]

Exercise Program: Neck

1a) Training Deep Neckflexors

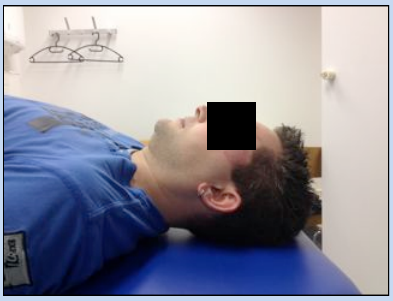
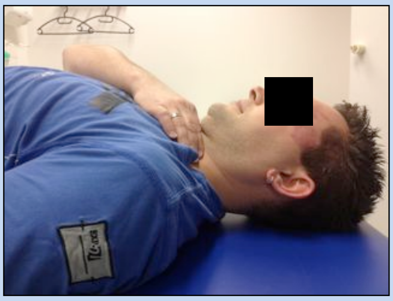

Starting Position **End Position**

Supine, leggs bend. No pillow underneath your head. Place one hand on your throat. Now start to look down towards your chin, the head follows with a small and slow movement (nodding). The back of your head should glide over the surface. Just move your head as far down as you start feeling some tention under your fingers. Keep this position for ten seconds.

Repetitions: 10 **Length: 10 Sec** **Frequency: 2/d**

1b) Training Deep Neckflexors

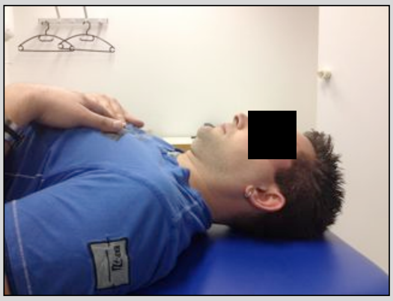
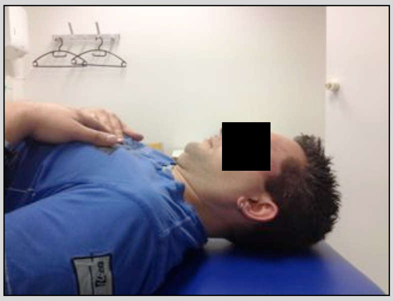

Starting Position **End Position**

Supine, hooklying, hands on your chest. No pillow underneath your head. Now tuck your chin in and lift your head about 1 cm. The position of your chin should be stable. You are allowed to have a 60-90 sec. break betweeen the repetitions. If you are able to do 12 repititions without a problem it is ok to increase up to 15 or even 20.

Repetitions: 3 Series of 12 (15 / 20) **Length: 1 Sec / Break 2 Sec** **Ferquency: 1/d**

2) Training Shouldergirdle

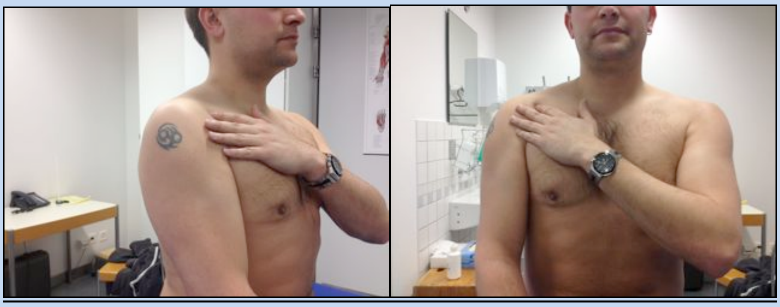
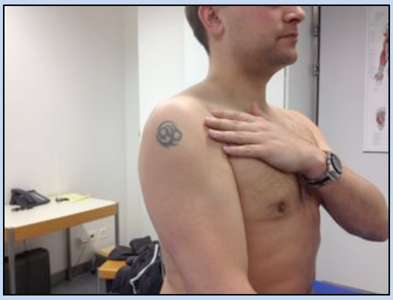

Starting Position **End Position**

Sitting on a chair, in front of a mirror. The left hand is placed on the front of your right shoulder. Your index finger is on the coracoid process. Now try to move the bone away from your hand in a longitudinal axis. Keep this position for ten seconds. Now do the same with the left shoulder. Later it is also ok to train both shoulders at the same time. If this exercise is to easy for you, put both hands on your thigh and, while you keep your shoulder in a good position, push down with your hands into your thigh.

Repititions: 10 **Length: 10 Sec** **Frequency: 2/d**

3) Head-Eye-Coordination

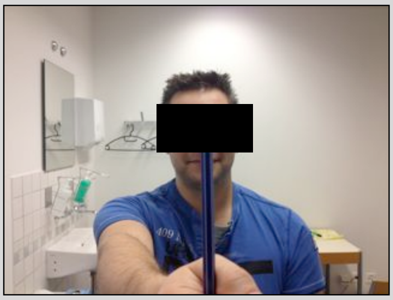 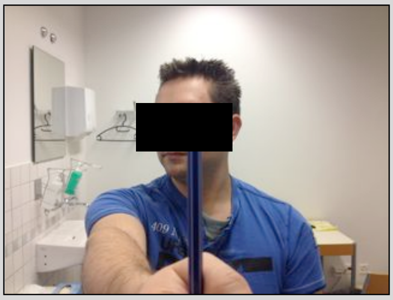 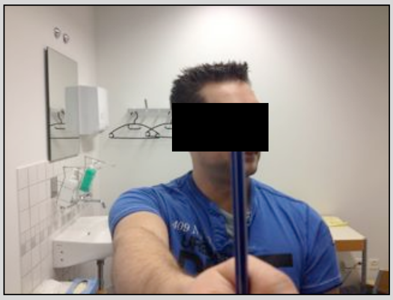

Starting Position	Position 1	Position 2

Sitting on a chair, you could either have a steady spot on the wall or hold a pen in your hand. If you chose a pen, put it into one hand and straighten the arm out, so that you can see the pen. Look at the pen. Now move your head and your eyes to the right and then back to the pen. Do the same to the left. If this exercise is to easy you can increase the level as follows.

- First move your eyes then the head to the target.
- Head and eyes move into opposite directions
- Do the exercise while standing

Repetitions: 3 Series of 5 to each side **Frequency: 1/d**

If you feel dizzy or light headed while exercising, please take a break!!
